# Impacts of sugarcane industrial effluent as an alternate source of irrigation on growth, chlorophyll contents and antioxidants of different canola varieties

**DOI:** 10.1038/s41598-023-49951-z

**Published:** 2024-01-22

**Authors:** Hafeez Ullah, Nosheen Noor Elahi, Muhammad Imtiaz, Muhammad Nadeem Shah, Mohammed Asiri, Mohammad Y. Alshahrani

**Affiliations:** 1 Institute of Botany, Bahauddin Zakariya University, Multan, 60000 Punjab Pakistan; 2 Soil and Environmental Biotechnology Division, National Institute for Biotechnology and Genetic Engineering (NIBGE), Faisalabad, Punjab Pakistan; 3 Department of Agriculture, Government College University, Lahore, Pakistan; 4 Department of Clinical Laboratory Sciences, College of Applied Medical Sciences, King Khalid University, Abha, Saudi Arabia

**Keywords:** Plant sciences, Plant physiology, Plant stress responses

## Abstract

The sugarcane industry often utilizes effluent for irrigation purposes; however, its intricate composition and elevated metal contaminants pose a potential risk of soil and crop contamination. Consequently, it is imperative to employ effective strategies to ensure the safe utilization of this resource for crop cultivation. One such strategy involves the dilution of sugarcane industry effluent. Dilution is a practical approach to mitigate its toxicity, minimizing its adverse impact on soil and crop health. That’s why the current study explored the best dilution of sugarcane industrial effluent (SW) for cultivating canola varieties. A total of 15 canola varieties were cultivated 0%, 20%, 40%, 60%, 80%, and 100% SW. Results showed that 60% SW Faisalabad Canola and Punjab Canola improved germination, shoot length, root length, shoot fresh and dry weight, root fresh and dry weight, and chlorophyll contents compared to other treatments and control. AARI Canola and CON-III showed poor growth and chlorophyll contents under 60%SW. Dunkled and Oscar cultivars showed moderate improvement in growth and chlorophyll contents under 60SW. The 60% SW can be recommended for maximum growth benefits in canola cultivars, specifically Faisalabad Canola and Punjab Canola. At 20SW, the root dry weight of Faisalabad Canola increased by 2.7%, while Punjab Canola increased by 3.4%. Canola showed the highest increase in POD activity compared to the control, with a 55.45% increase, followed by Sandal Canola, with a 43.26% increase. However, additional field-level experiments are needed to determine the best cultivars suitable for optimal growth under 80SW and 60SW irrigation conditions.

## Introduction

The scarcity of water resources is a major challenge in arid and semi-arid areas^1–3^, which limits agricultural productivity^4–6^ and poses a threat to food security^7,8^. In these regions, irrigation is essential for crop growth and development, but limited water resources pose a challenge^9,10^. In this context, sugarcane industrial effluent can be considered a potential alternative source of irrigation water, which can meet the water demands of crops and alleviate the scarcity of irrigation water^11^.

Sugarcane industrial effluent is a byproduct of the sugarcane industry, which is rich in nutrients and organic matter, making it a good source of irrigation water^12^. On average, sugarcane effluent contains pH between 6.5 and 7.0, total suspended solids 110–104 mg dm^3^, total dissolved solids 400–1.650 mg dm^−3^, and dissolved oxygen 2.52–2.95 mg dm^−3^^13^. Raza et al.^14^ reported 93% water, 19.5 g L^−1^ organic matter, 3.3% nitrogen, 1190 mg L^−1^ phosphorus, 120 mg L^−1^ potassium 120 mg L^−1^, pH between 3.5–5 and 0.328 dS m^−1^ electrical conductivity. Being enriched in nutrients, sugarcane industrial effluent can be used for plant growth and development. It can be an alternative source of fertilizer in areas where other sources of fertilizers are scarce^15^. However, it should be noted that using sugarcane industrial effluent for irrigation requires careful management. The effluent may contain high levels of salts and heavy metals, which can accumulate in the soil and affect crop growth and quality^16^.

Sugarcane vinasse, known for its complex chemical composition, poses a significant pollution threat to areas near sugarcane industries. A distillery producing 100 m^3^ of ethanol daily discharges 1300 m^3^ of sugarcane vinasse with a high biochemical oxygen demand (BOD), equivalent to the domestic sewage output of a community of 1.5 lakh people. Similarly, spent wash from distilleries negatively affects plant growth, reducing seed germination and soil fertility due to its high concentration of organic and inorganic pollutants, including heavy metals^14^. Furthermore, heavy metals in the effluent, such as lead, cadmium, and mercury, can also cause plant toxicity. These heavy metals can accumulate in the plant tissues, reducing growth and yield^17^. Toxicity of heavy metals can cause oxidative stress, damaging plant cells and reducing photosynthesis^18^. Dilution of sugarcane industrial effluent can be a viable solution to reduce the negative impacts of high salts and heavy metals on plant growth. However, it is important to note that the optimal dilution rate may vary depending on the crop species and environmental conditions^19^.

Canola is an important crop as it is a significant source of edible oil and protein-rich animal feed^20^. Its oil is considered a healthy cooking oil due to its low saturated fat content and high levels of unsaturated fatty acids, such as omega-3 and omega-6 fatty acids, linked to numerous health benefits^21,22^. While limited literature is available on the use of diluted sugarcane industrial effluent for canola cultivation, current study was conducted to investigate the appropriate dilution rate of sugarcane industrial effluent (SW) for canola cultivation. This study covers the knowledge gap regarding using SW appropriate dilution for better canola cultivation. Also, study findings will help growers identify the tolerant, moderate, and susceptible varieties of canola for cultivation using SW.

## Material and methods

A laboratory hydroponic experiment was conducted to determine the suitability of canola varieties for cultivation using diluted sugarcane industrial effluent (SW) as an irrigation source. The experiment aimed to identify canola varieties that were resistant, moderately resistant, and sensitive to the different levels of SW dilution.

### Seeds collection

Fifteen canola varieties were procured from the Ayub Agriculture Research Institute and included in the laboratory hydroponic experiment. The cultivars used in the study were Dunkled, Super Raya, Rainbow, Punjab Canola, Oscar, Legend, AARI Canola, AC Exul, CON-II, CON-III, Cyclone, Faisalabad Canola, Super Canola, Sandal Canola, and Shiralee. Before beginning the experiment, manual screening was done to separate broken and damaged seeds.

### Sugarcane industrial effluent

A sample of sugarcane industrial effluent (SW) was obtained from a local sugar factory, and its properties were analyzed in the laboratory before the experiment. The results of the analysis are presented in Table 1. Serial dilutions were prepared by mixing the SW with tap water based on the characteristics of the sugarcane industrial effluent. The concentrations of SW used in the experiment included a control group with no SW and 1000 ml of tap water, as well as 20% (200 ml of SW and 800 ml of tap water), 40% (400 ml of SW and 600 ml of tap water), 60% (600 ml of SW and 400 ml of tap water), 80% (800 ml of SW and 200 ml of tap water), and 100% (1000 ml of SW and no tap water). These concentrations were used for testing the growth of canola varieties under different levels of SW dilution. To ensure the accuracy of the experiment, all concentrations of SW were mixed and prepared under strict laboratory conditions.

**Table 1:** Pre-experimental characteristics of sugarcane industrial effluent (SW).

Attribute	Unit	Value
pH	–	6.51
EC	dS/m	3.66
Nitrogen	mg L^−1^	10.14
Phosphorus		12.94
Potassium		166
Magnesium		17.52
Calcium		42.41
Chloride		3.5
Carbonates	meq L^−1^	0.31
Bicarbonates		2.67
Cadmium	mg L^−1^	Not detected
Lead		6.83
Arsenic		Not detected
Chromium		0.71

### Treatment plan

The hydroponic experiment was conducted using a factorial completely randomized design, comprising six different levels of sugarcane industrial effluent (SW) (control, 20, 40, 60, 80, and 100%) and 15 canola varieties (Dunkled, Super Raya, Rainbow, Punjab Canola, Oscar, Legend, AARI Canola, AC Exul, CON-II, CON-III, Cyclone, Faisalabad Canola, Super Canola, Sandal Canola, and Shiralee). A total of 90 treatment combinations were tested in three replicates to ensure the reliability and validity of the results.

### Sowing and incubation conditions

Initially, ten seeds were sown in sterilized petri dishes in three replicates. To maintain sterilized conditions, the seeds were placed between sterilized filter papers. The petri dishes were then placed in an incubator at a temperature of 25 °C ± 3 °C for the duration of the experiment. To ensure that the seeds could germinate and grow under optimal conditions, the humidity in the incubator was maintained at 70% throughout the experiment.

### Data collection and harvesting

In this experiment, the growth parameters of canola plants were assessed after 15 days of sowing. The germination percentage was calculated by counting the number of seeds germinating on the 3rd and 7th day after sowing. After 15 days, three healthy seedlings were harvested from each petri dish for the first harvest. The root and shoot lengths were measured using a standard scale, and the fresh weights of the root and shoot were taken using an analytical-grade balance. The shoot and root samples were then oven-dried for 48 h until the constant weight at 65 °C to determine their dry weights. They were subsequently re-weighed on an analytical-grade balance.

### Transplantation

After the first harvest, the seedlings were transplanted into plastic cups with a depth of 5 inches and a diameter of 3 inches. The shoots of the seedlings were supported using a rolled filter paper. The experiment was carried out for 30 days from the day of sowing to monitor the growth of the canola plants.

### Harvesting for antioxidants

The second harvest was conducted after 30 days of sowing, during which three healthy seedlings were collected from each petri dish. Fresh leaf samples were taken to analyze the levels of chlorophyll contents and antioxidants using a spectrophotometer.

### Chlorophyll contents

For the assessment of chlorophyll contents in the leaves, 80% acetone solution was used during the grinding of samples. After filtration the final volume was adjusted to 15 ml with acetone and absorbance was noted at 663 and 645 nm wavelength on UV-spectrophotometer. Final values of chlorophyll a, b and total were computed using the eq.

$$\text{Chlorophyll a }\left(\frac{{\text{mg}}}{{\text{g}}}\right)=\frac{\left(12.7\times {\text{A}}663\right)-\left(2.69\times {\text{A}}645\right)\times {\text{V}}}{1000\times {\text{W}}}$$

$$\text{Chlorophyll b }\left(\frac{{\text{mg}}}{{\text{g}}}\right)=\frac{\left(22.9\times {\text{A}}645\right)-\left(4.68\times {\text{A}}663\right)\times {\text{V}}}{1000\times {\text{W}}}$$

$$\text{Total Chlorophyll }\left(\frac{{\text{mg}}}{{\text{g}}}\right)= 20.2\left(\mathrm{OD }645\right)+8.02\left(\mathrm{OD }663\right)\times {\text{V}}/1000 ({\text{W}})$$

### POD assay

To perform the peroxidase (POD) assay on fresh plant leaves, the leaves were first collected and washed with distilled water to remove any dirt or debris. Then, they were dried using filter paper. The dried leaves were ground in a mortar and pestle with 50 mM phosphate buffer (pH 7.0) to make a homogenate. The supernatant was obtained by centrifuging the homogenate at 10,000×*g* for 10 min at 4 °C. The POD enzyme was extracted from the supernatant. To assay the POD activity, 0.1 mL of the POD supernatant was mixed with 1.9 mL of the assay buffer (50 mM phosphate buffer, pH 7.0) and 0.1 mL of guaiacol as the substrate. The reaction mixture was incubated at room temperature for 5 min and stopped by adding 1.0 mL of 1 M H_2_SO_4_. The optical density was immediately measured at 470 nm using a spectrophotometer. The POD activity was calculated using a standard curve^23^.

### CAT assay

The catalase (CAT) enzyme supernatant was centrifuged at 10,000 × g for 10 min at 4 °C. For the assay, 0.1 mL of the CAT supernatant was mixed with 1.9 mL of the assay buffer (50 mM phosphate buffer, pH 7.0) and 0.1 mL of hydrogen peroxide (H_2_O_2_) as the substrate. The decrease in H_2_O_2_ concentration was monitored spectrophotometrically by measuring the decrease in absorbance at 240 nm over time^24^.

### SOD assay

The superoxide dismutase (SOD) enzyme was extracted from the homogenate by centrifugation at 10,000×*g* for 10 min at 4 °C. The SOD activity was determined spectrophotometrically based on the inhibition of the photochemical reaction of nitro blue tetrazolium (NBT) with superoxide radicals. The reaction mixture contained 0.1 ml of the SOD supernatant, 1.9 mL of the assay buffer (e.g., 50 mM phosphate buffer, pH 7.0), and 0.1 ml of a solution containing NBT and riboflavin. The increase in absorbance at 560 nm was measured over time^25^.

### MDA assay

The concentration of MDA was determined using a colorimetric assay based on the reaction of MDA with thiobarbituric acid (TBA) to form a pink-colored complex. A reaction mixture containing 0.1 mL of the extracted sample and 1.9 mL of a reaction buffer (e.g., 0.67% (w/v) TBA in 20% (v/v) acetic acid) was heated at 95–100 °C for 20 min. The absorbance of the reaction mixture was measured at 532 nm^26^.

### Statistical analyses

A standard statistical procedure was followed to analyze the data^27^. The statistical analysis involved a two-way analysis of variance (ANOVA) with Fisher's least significant difference (LSD) test, performed at a significance level of p ≤ 0.05, to compare the treatments. The data analysis and graph creation were done using OriginPro2021 software^28^.

### Ethics approval and consent to participate

We all declare that manuscript reporting studies do not involve any human participants, human data, or human tissue. So, it is not applicable.

### Study protocol must comply with relevant institutional, national, and international guidelines and legislation

Our experiment follows the with relevant institutional, national, and international guidelines and legislation.

## Results

**Figure 1:**
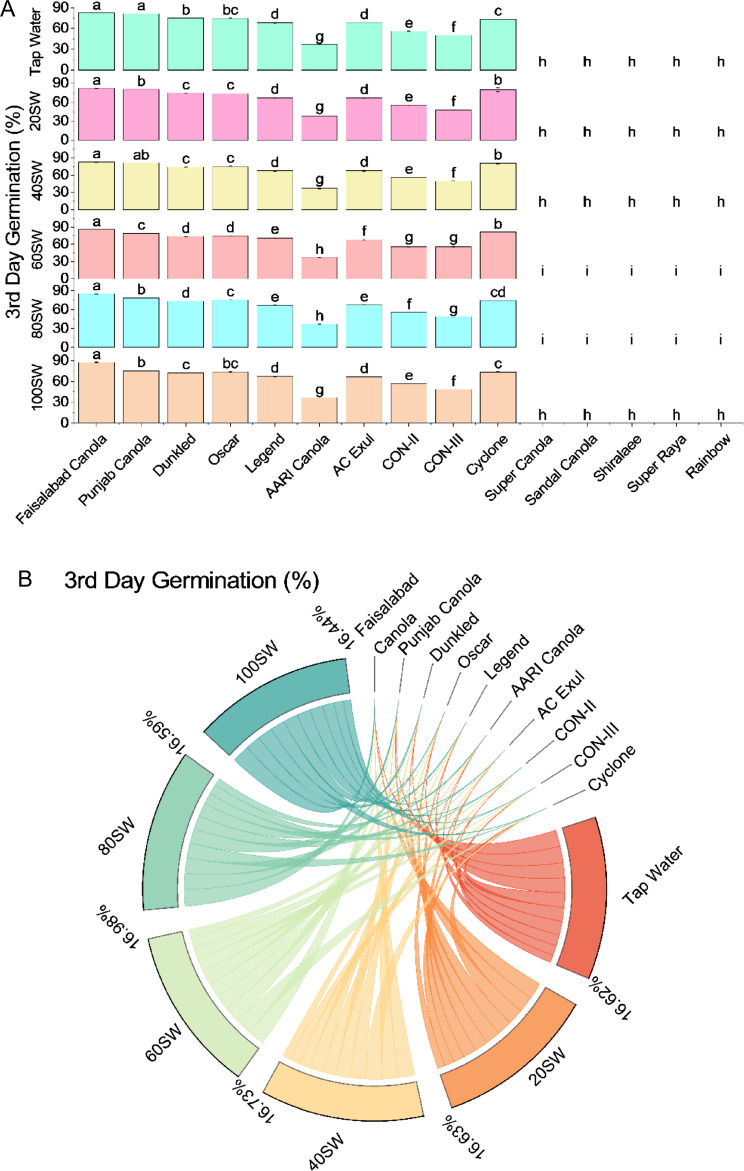
Effects of different sugarcane industrial effluent (SW) levels on 3rd day germination. Bars are means of three replicates ± SE (**A**). Different letters showed significant changes compared with Fisher LSD; p ≤ 0.05. Average percentage contribution of each SW level for improvement in 3rd day germination is provided in Chord diagram (**B**).

The highest germination percentage was observed in Faisalabad Canola with tap water irrigation, followed closely by Punjab Canola with tap water irrigation. However, in irrigation treatments, Super Canola, Sandal Canola, Shiralaee, Super Raya, and Rainbow showed no germination. When exposed to 20% SW irrigation, Faisalabad Canola and Punjab Canola still had high germination percentages. Following Dunkled and Oscar, Cyclone had a relatively high germination percentage. AARI Canola showed the lowest germination percentage in this treatment. When exposed to 40% SW irrigation, Faisalabad Canola maintained the highest germination percentage, followed by Punjab Canola and Cyclone. The rest of the varieties had lower germination percentages. At 60% SW irrigation, Faisalabad Canola had the highest germination percentage again, but Punjab Canola significantly decreased germination. Dunkled and Oscar had similar germination percentages, while AARI Canola had the lowest. At 80% SW irrigation, Faisalabad Canola had the highest germination percentage, followed by Oscar and AC Exul. Punjab Canola had a significant decrease in germination percentage. At the same time, Super Canola, Sandal Canola, Shiralaee, Super Raya, and Rainbow showed no germination. Finally, at 100% SW irrigation, Faisalabad Canola had the highest germination percentage, while Punjab Canola had the lowest. Dunkled and Oscar also had relatively low germination percentages, while AARI Canola had the lowest (Fig. 1).

**Figure 2:**
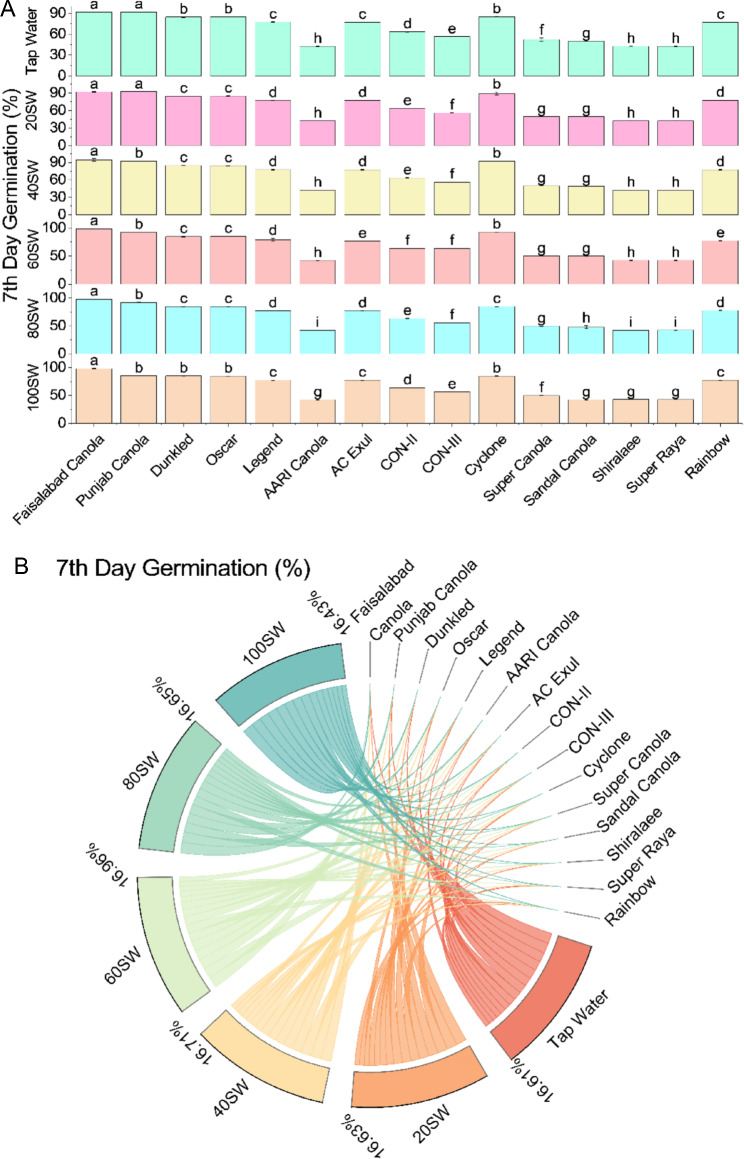
Effects of different sugarcane industrial effluent (SW) levels on 7th day germination. Bars are means of three replicates ± SE (**A**). Different letters showed significant changes compared with Fisher LSD; p ≤ 0.05. Average percentage contribution of each SW level for improvement in 7th day germination is provided in Chord diagram (**B**).

The results showed that the 7th day germination percentage varied significantly among the canola varieties at different levels of SW irrigation. Among the tested varieties, Faisalabad Canola and Punjab Canola exhibited the highest germination percentage in all treatments, followed by Dunkled, Oscar, Cyclone, and Legend. However, AARI Canola, AC Exul, CON-II, CON-III, Super Canola, Sandal Canola, Shiralaee, and Super Raya showed relatively lower germination percentages in all treatments, particularly under 100% SW irrigation. In detail, at 20% SW irrigation, Faisalabad Canola and Punjab Canola exhibited the highest germination percentage (91.94% and 92.38%, respectively). In contrast, AARI Canola and Shiralaee showed the lowest germination percentage (42.52% and 42.41%, respectively). At 40% SW irrigation, Faisalabad Canola exhibited the highest germination percentage (94.40%), followed by Punjab Canola (92.20%). AARI Canola and Shiralaee showed the lowest germination percentage (42.39% and 42.37%, respectively). At 60% SW irrigation, Faisalabad Canola exhibited the highest germination percentage (98.51%), followed by Punjab Canola (92.53%). AARI Canola showed the lowest germination percentage (42.29%). At 80% SW irrigation, Faisalabad Canola exhibited the highest germination percentage (98.17%), followed by Punjab Canola (92.27%). AARI Canola and Sandal Canola showed the lowest germination percentage (42.29% and 47.69%, respectively). At 100% SW irrigation, Faisalabad Canola exhibited the highest germination percentage (98.36%), whereas Punjab Canola showed the lowest germination percentage (85.26%). AARI Canola, Sandal Canola, Super Canola, and Super Raya showed the lowest germination percentages among all varieties, with values ranging from 42.13 to 49.74% (Fig. 2).

In tap water, the highest shoot length was observed for Faisalabad Canola with a value of 9.42 cm, followed by Punjab Canola with 9.34 cm. The lowest shoot length in tap water was observed for CON-III with a value of 3.19 cm. The other canola varieties showed shoot lengths ranging from 4.32 to 7.34 cm. When grown in 20% sewage water, Faisalabad Canola showed the highest shoot length with a value of 9.61 cm. In contrast, the lowest shoot length was observed for CON-III with a value of 3.96 cm.

The shoot lengths of other canola varieties ranged from 4.35 to 7.36 cm. In 40% sewage water, Faisalabad Canola again showed the highest shoot length with a value of 9.65 cm. In contrast, AARI Canola showed the lowest shoot length with a value of 3.96 cm. The shoot lengths of other canola varieties ranged from 6.04 to 8.35 cm. When grown in 60% sewage water, Faisalabad Canola showed the highest shoot length with a value of 10.11 cm.

**Figure 3:**
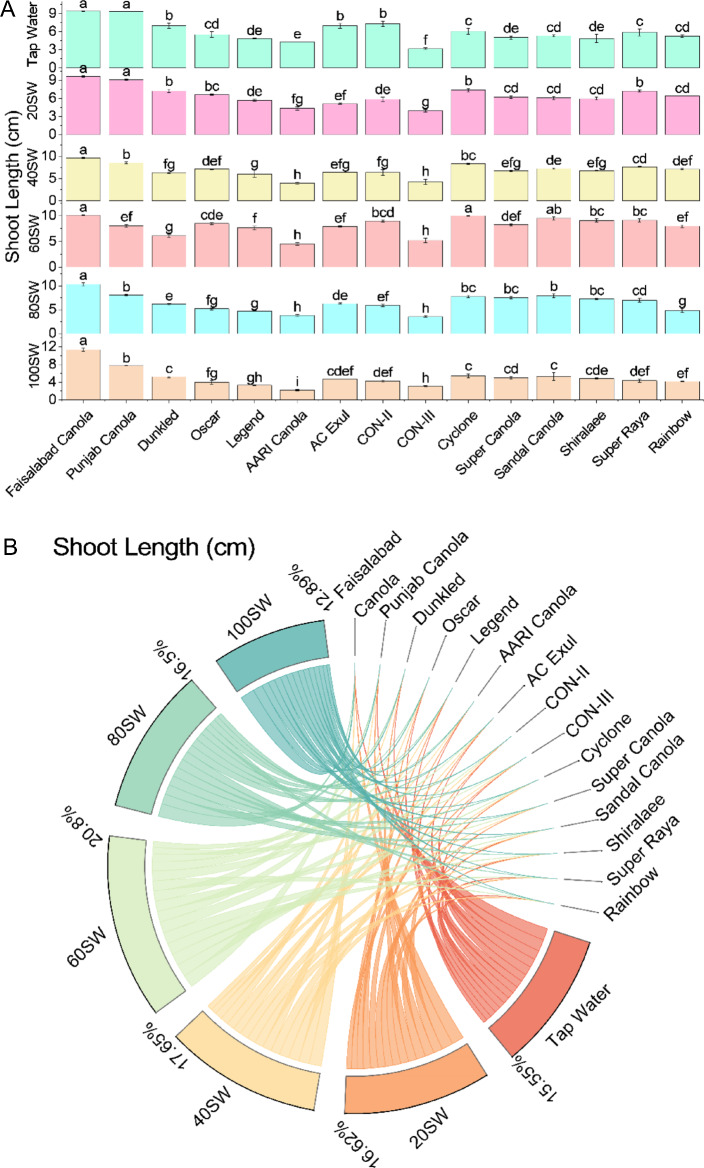
Effects of different sugarcane industrial effluent (SW) levels on shoot length. Bars are means of three replicates ± SE (**A**). Different letters showed significant changes compared with Fisher LSD; p ≤ 0.05. Average percentage contribution of each SW level for improvement in shoot length is provided in Chord diagram (**B**).

In contrast, Punjab Canola showed the lowest shoot length with a value of 8.08 cm. The shoot lengths of other canola varieties ranged from 4.51 to 9.98 cm. In 80% sewage water, the highest shoot length was observed for Faisalabad Canola with a value of 10.29 cm. In contrast, the lowest shoot length was observed for Legend with a value of 4.72 cm. The shoot lengths of other canola varieties ranged from 3.77 to 7.88 cm. In 100% sewage water, Faisalabad Canola showed the highest shoot length with a value of 11.36 cm, while Oscar showed the lowest shoot length with a value of 3.97 cm. The shoot lengths of other canola varieties ranged from 2.24 to 5.27 cm (Fig. 3).

**Figure 4:**
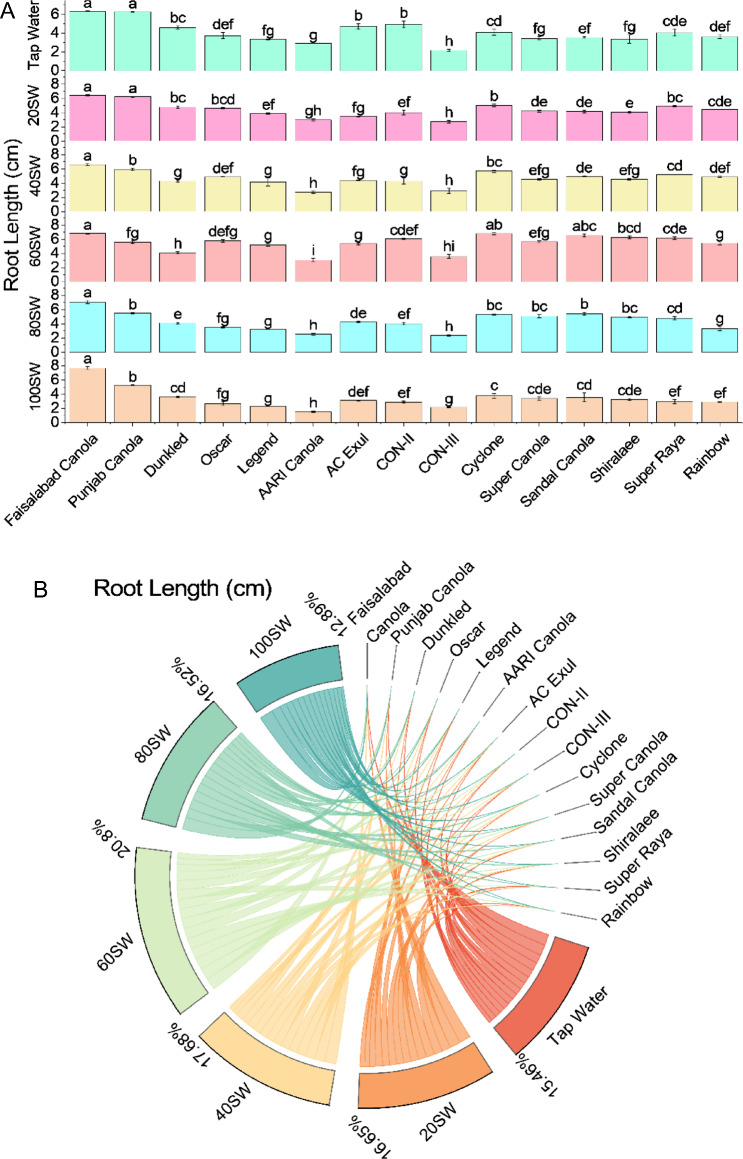
Effects of different sugarcane industrial effluent (SW) levels on root length. Bars are means of three replicates ± SE (**A**). Different letters showed significant changes compared with Fisher LSD; p ≤ 0.05. Average percentage contribution of each SW level for improvement in root length is provided in Chord diagram (**B**).

Regarding root length, Faisalabad Canola had the longest measurement at 6.33 cm with tap water irrigation. This was slightly longer than Punjab Canola's root length of 6.27 cm. Dunkled, Oscar, and Legend had shorter root lengths of 4.59 cm, 3.73 cm, and 3.34 cm, respectively. The remaining varieties (AARI Canola, AC Exul, CON-II, CON-III, Cyclone, Super Canola, Sandal Canola, Shiralaee, Super Raya, and Rainbow) had root lengths ranging from 2.94 to 4.95 cm. When the soil water content was at 20%, Faisalabad Canola still had the longest root length at 6.43 cm, followed by Punjab Canola at 6.22706 cm. Dunkled, Oscar, and Legend had root lengths of 4.78 cm, 4.63 cm, and 3.86 cm, respectively. The remaining varieties had root lengths ranging from 2.97 to 5.00 cm. At 40% SWC, Faisalabad Canola had the longest root length at 6.58 cm, while Punjab Canola had a slightly shorter root length of 5.91 cm. Dunkled, Oscar, and Legend had root lengths of 4.29 cm, 4.89311 cm, and 4.15 cm, respectively. The other varieties had root lengths ranging from 2.70 to 5.66 cm. When SW was 60%, Faisalabad Canola again had the longest root length at 6.82 cm, while Punjab Canola had a slightly shorter root length of 5.57 cm. Dunkled had a root length of 4.0892 cm, followed by Oscar and Legend with 5.76 cm and 5.21 cm respectively. The remaining varieties had root lengths ranging from 3.06 to 6.79 cm. At 80% SWC, Faisalabad Canola still had the longest root length at 7.04 cm, while Punjab Canola had a slightly shorter root length of 5.49 cm. Dunkled had a root length of 4.11 cm, followed by Oscar and Legend with 3.51 cm and 3.20 cm, respectively. The other varieties had root lengths ranging from 2.53 to 5.39 cm. Finally, at 100% SWC, Faisalabad Canola had the longest root length at 7.66163 cm. In contrast, Punjab Canola had a slightly shorter root length of 5.26 cm. AARI Canola had the shortest root length at 1.53 cm, while the remaining varieties had root lengths ranging from 2.66 to 3.77 cm. All root length measurements were rounded to two decimal places (Fig. 4).

At tap water irrigation level, the highest seedling fresh weight was observed for Faisalabad Canola with 0.07353 g and the lowest was for CON-III with 0.02127 g. Moderate seedling fresh weights were observed for Dunkled, Oscar, and AARI Canola with values ranging from 0.03166 to 0.04981 g. In contrast, the remaining varieties showed susceptible seedling fresh weight with values ranging from 0.03179 to 0.05568 g. With the increase in sewage water irrigation level from 20 to 100%, the seedling fresh weight of most canola varieties decreased except for a few that showed a moderate increase or remained the same. For instance, Faisalabad Canola, which had the highest seedling fresh weight in tap water, showed a moderate increase in shoot fresh weight with 0.07422 g at 20% sewage water irrigation level but then showed a decreasing trend with a further increase in sewage water level.

**Figure 5:**
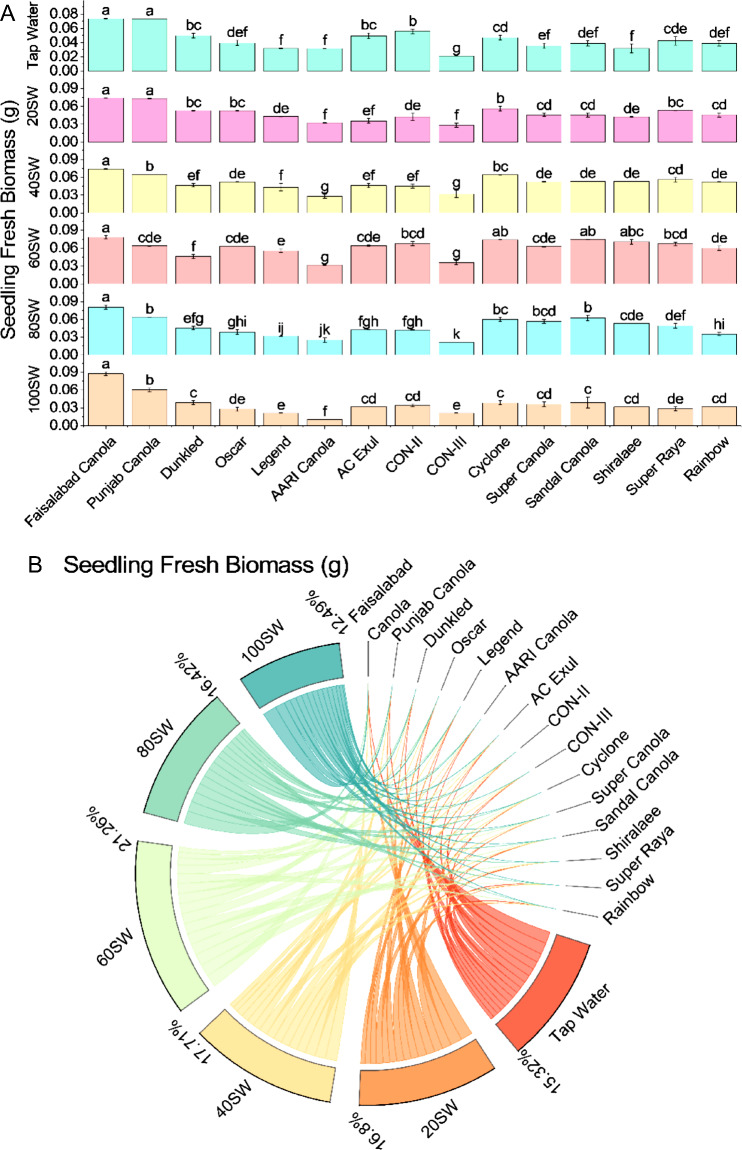
Effects of different sugarcane industrial effluent (SW) levels on seedling fresh biomass. Bars are means of three replicates ± SE (**A**). Different letters showed significant changes compared with Fisher LSD; p ≤ 0.05. Average percentage contribution of each SW level for improvement in seedling fresh biomass is provided in Chord diagram (**B**).

Similarly, Punjab Canola had a moderate shoot fresh weight in tap water. It showed a slight decrease in seedling fresh weight with 0.07325 g at 20% sewage water irrigation level and a more significant decrease with 0.06012 g at 100% sewage water irrigation level. On the other hand, Cyclone, a susceptible variety at tap water, showed a moderate increase in seedling fresh weight with 0.05611 g at 20% sewage water irrigation level and the highest seedling fresh weight among all varieties at 60% and 80% sewage water irrigation levels with 0.0741 g and 0.05975 g, respectively. However, Cyclone also showed a decreasing trend in seedling fresh weight at 100% sewage water irrigation level with 0.03841 g. AC Exul, another susceptible variety at tap water, showed a moderate increase in seedling fresh weight with 0.0354 g at 20% sewage water irrigation level and remained almost the same at 40% and 60% sewage water irrigation levels, then showed a decreasing trend with further increase in sewage water level (Fig. 5).

**Figure 6:**
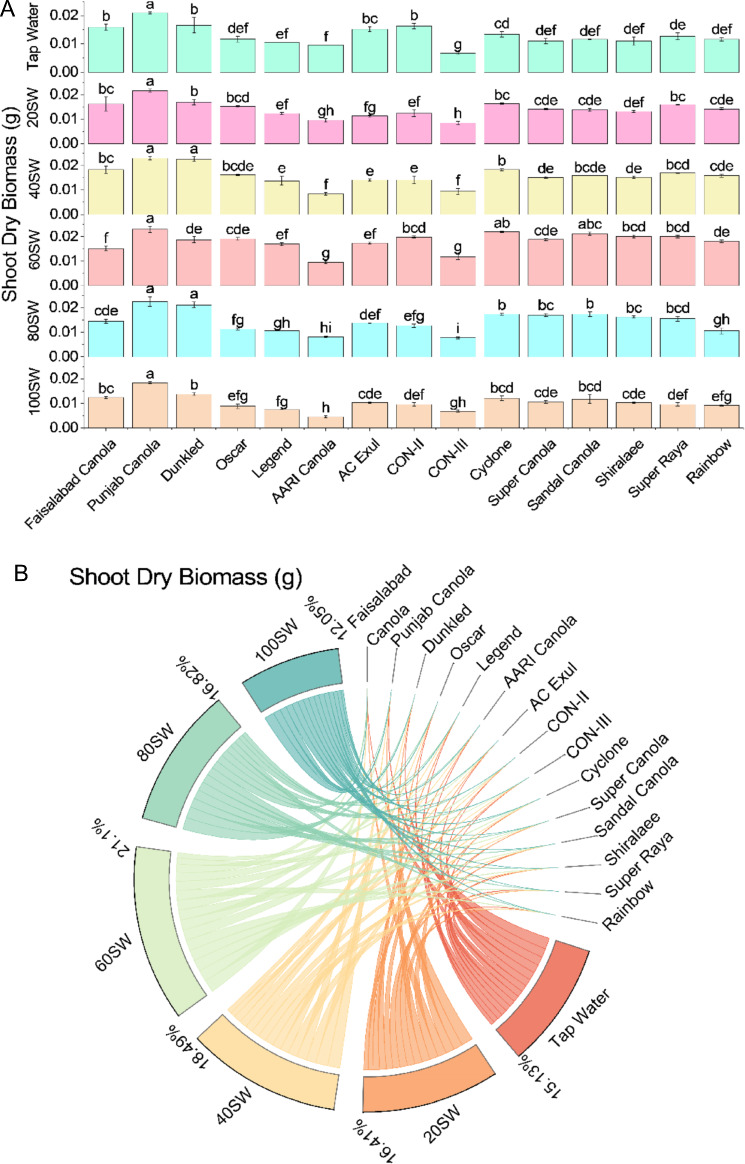
Effects of different sugarcane industrial effluent (SW) levels on shoot dry biomass. Bars are means of three replicates ± SE (**A**). Different letters showed significant changes compared with Fisher LSD; p ≤ 0.05. Average percentage contribution of each SW level for improvement in shoot dry biomass is provided in Chord diagram (**B**).

Based on the table, the shoot dry weight of canola varieties generally increased as the soil water regime increased from tap water to 100SW. However, the extent of increase varied among different varieties. For instance, Faisalabad Canola showed an increase in shoot dry weight from 0.0159 g under tap water to 0.01834 g under 40SW. However, it then showed a decrease to 0.01451 g under 80SW. On the other hand, Punjab Canola showed a consistent increase in shoot dry weight from 0.02096 g under tap water to 0.02306 g under 40SW. Then it showed a slight decrease to 0.02251 g under 80SW. Generally, the highest shoot dry weight values were observed at 60SW and 80SW for most canola varieties. For example, Dunkled increased shoot dry weight from 0.0166 g under tap water to 0.02199 g under 60SW. In comparison, Punjab Canola showed an increase from 0.02096 g under tap water to 0.02251 g under 80SW. It is worth noting that some canola varieties, such as CON-III, decreased shoot dry weight as the soil water regime increased. For example, CON-III decreased shoot dry weight from 0.00672 g under tap water to 0.00776 g under 80SW (Fig. 6).

**Figure 7:**
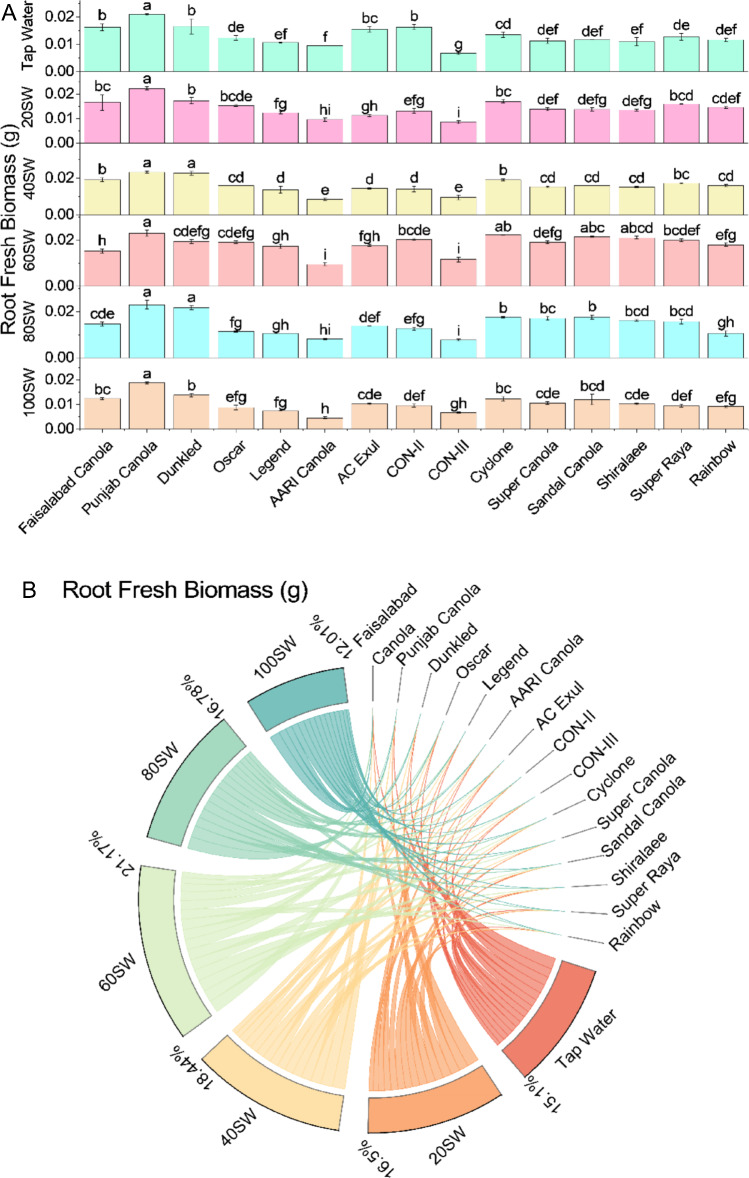
Effects of different sugarcane industrial effluent (SW) levels on root fresh biomass. Bars are means of three replicates ± SE (**A**). Different letters showed significant changes compared with Fisher LSD; p ≤ 0.05. Average percentage contribution of each SW level for improvement in root fresh biomass is provided in Chord diagram (**B**).

Results showed that at 20SW, most of the varieties showed an increase in root fresh biomass compared to tap water. Faisalabad Canola showed an increase of 1.2%, Punjab Canola showed an increase of 6.5%, and Dunkled increased 4.4%, while Oscar and Legend showed a decrease of 4.4% and 0.8%, respectively. Similarly, at 40SW, Punjab Canola and Dunkled showed an increase of 11.7% and 36.7%, respectively, while Oscar and Legend showed a decrease of 22.3% and 2.5%, respectively, compared to tap water. At 60SW, Dunkled, Cyclone, and Sandal Canola showed an increase in root fresh biomass by 17.2%, 65.2%, and 83.5%, respectively, compared to tap water, while Legend and Oscar showed a decrease of 6.5% and 0.9%, respectively. At 80SW, most of the varieties showed a decrease in root fresh biomass compared to tap water, except for Dunkled, which showed an increase of 31.1%. Punjab Canola and Sandal Canola showed a decrease of 2.3% and 3.3%, respectively, while Legend and Oscar showed a decrease of 4.4% and 7.8%, respectively. At 100SW, most of the varieties showed a significant decrease in root fresh biomass compared to tap water, ranging from 8.8 to 53.3%, except for Dunkled, which only decreased by 16.4% (Fig. 7).

**Figure 8:**
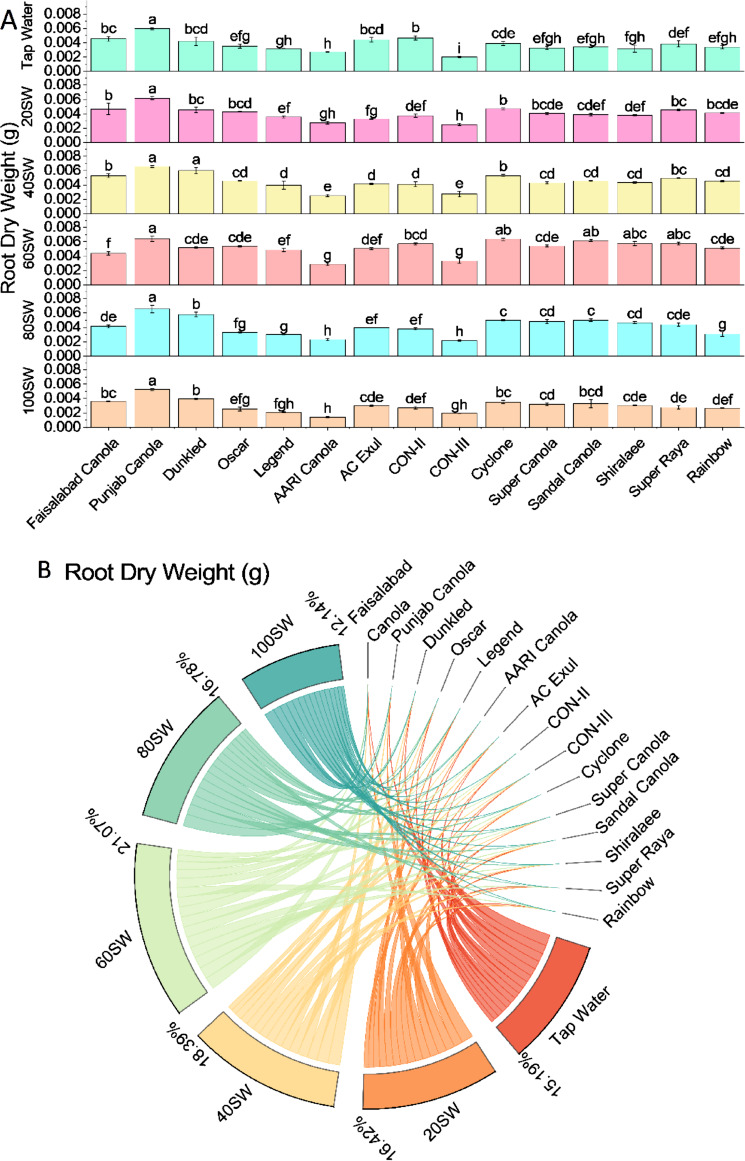
Effects of different sugarcane industrial effluent (SW) levels on root dry biomass. Bars are means of three replicates ± SE (**A**). Different letters showed significant changes compared with Fisher LSD; p ≤ 0.05. Average percentage contribution of each SW level for improvement in root dry biomass is provided in Chord diagram (**B**).

The root dry weight of various canola cultivars was measured under different irrigation levels (tap water, 20SW, 40SW, 60SW, 80SW, and 100SW). The results are presented in grams (g). Compared to tap water, the root dry weight of canola cultivars generally decreased under different irrigation levels. At 20SW, the root dry weight of Faisalabad Canola increased by 2.7%, while Punjab Canola increased by 3.4%. On the other hand, Oscar and Legend had a 21.4% and 16.1% decrease in root dry weight, respectively. At 40SW, the root dry weight of Punjab Canola increased by 14.7%. In contrast, Oscar and Legend had a decrease in root dry weight by 23.3% and 20.3%, respectively. At 60SW, Punjab Canola showed an increase in root dry weight by 40.9%, while Oscar and Legend decreased root dry weight by 5.5% and 8.5%, respectively. At 80SW, Punjab Canola showed an increase in root dry weight by 44.2%, while Oscar and Legend decreased root dry weight by 35.6% and 39.9%, respectively. At 100SW, Punjab Canola showed an increase in root dry weight by 15.3%, while Oscar and Legend decreased root dry weight by 28.6% and 34.0%, respectively (Fig. 8).

In 20SW, Faisalabad Canola had the highest POD activity, while Dunkled had the lowest. Super canola showed the highest increase in POD activity compared to the control, with a 36.12% increase, followed by Sandal Canola with a 25.77% increase. On the other hand, CON-III exhibited the highest decrease in POD activity compared to the control, with a reduction of 16.67%. At 40SW, Faisalabad Canola exhibited the highest POD activity again, while Punjab Canola had the lowest. Super canola showed the highest increase in POD activity compared to the control, with a 55.45% increase, followed by Sandal Canola with a 43.26% increase. Dunkled exhibited the highest decrease in POD activity compared to the control, with a reduction of 33.97%. Under 60SW, Faisalabad Canola had the highest POD activity, while Punjab Canola had the lowest. Super canola showed the highest increase in POD activity compared to the control, with a 60.44% increase, followed by Sandal Canola with a 57.45% increase. Dunkled exhibited the highest decrease in POD activity compared to the control, with a reduction of 38.93%. For 80SW, Faisalabad Canola had the highest POD activity, while Punjab Canola had the lowest. Super canola showed the highest increase in POD activity compared to the control, with a 70.22% increase, followed by Sandal Canola with a 68.38% increase. Dunkled exhibited the highest decrease in POD activity compared to the control, with a reduction of 42.92%. At 100SW, Faisalabad Canola again had the highest POD activity, while Punjab Canola had the lowest. Super canola showed the highest increase in POD activity compared to the control, with a 77.17% increase, followed by Sandal Canola with a 66.08% increase. Dunkled and CON-III were the most susceptible, with significant reductions in POD activity by 35.03% and 33.09%, respectively, compared to the control.

The Faisalabad Canola variety showed no significant difference in CAT activity compared to tap water. The Punjab Canola variety showed a slight increase in CAT activity at 20SW (10.71%) and a slight decrease at 60SW (− 3.45%). The Dunkled variety showed a slight decrease in CAT activity at 20SW (− 8.00%) and a slight increase at 60SW (7.41%). The Oscar variety showed a significant increase in CAT activity at all SW levels, ranging from 25.71% at 40SW to 110.34% at 80SW compared to tap water. The Legend variety also showed a significant increase in CAT activity at all SW levels, ranging from 3.85% at 40SW to 100.00% at 60SW. The AARI Canola and AC Exul varieties showed significant increases in CAT activity at all SW levels, ranging from 7.14 to 52.63% and 14.29–52.63%, respectively, compared to tap water. The CON-II variety showed a slight decrease in CAT activity at 60SW (-3.45%) and a significant decrease at 80SW (-27.59%) compared to tap water. The CON-III variety significantly increased CAT activity at all SW levels, ranging from 6.67% at 20SW to 46.67% at 80SW. The Cyclone variety showed a significant increase in CAT activity at all SW levels, ranging from 35.90% at 20SW to 61.54% at 60SW compared to tap water. The Super Canola and Sandal Canola varieties showed significant increases in CAT activity at all SW levels, ranging from 16.67 to 52.63% and 11.11–61.11%, respectively, compared to tap water. The Shiralaee variety significantly increased CAT activity at all SW levels, ranging from 10.53% at 20SW to 15.38% at 60SW compared to tap water. The Super Raya and Rainbow varieties showed a significant increase in CAT activity at 20SW (4.00% and 8.00%, respectively) compared to tap water, but no significant difference at other SW levels (Table 2).

**Table 2:** Effect of treatments on POD and CAT of different canola cultivars.

Attribute	Varieties	Tap water	20SW	40SW	60SW	80SW	100SW
POD (U mg^−1^ protein)	Faisalabad Canola	1.92h	1.91hi	1.87g	1.84gh	1.83g	1.82g
	Punjab Canola	1.47ij	1.46j	1.43h	1.41k	1.37i	1.35i
	Dunkled	1.31j	1.25k	1.23i	1.21l	1.20i	1.19i
	Oscar	1.52i	1.51j	1.51h	1.48jk	1.56h	1.59h
	Legend	1.91h	2.04h	1.78g	1.62ij	2.08f	1.80g
	AARI Canola	2.04h	1.78i	1.72g	1.68hi	2.34e	2.36f
	AC Exul	2.44f	2.46f	2.42e	2.39cd	2.51de	2.52ef
	CON-II	2.59ef	2.58ef	2.58de	2.56c	2.65d	2.67de
	CON-III	2.74de	2.72e	2.72cd	2.00fg	2.52de	2.79d
	Cyclone	2.90d	2.94d	2.87c	2.83b	2.99c	3.01c
	Super Canola	3.68a	3.69a	3.26ab	2.31de	3.13bc	3.19b
	Sandal Canola	3.22c	3.22c	3.23ab	3.23a	3.27b	3.27b
	Shiralaee	3.44b	3.42b	3.36a	3.32a	3.48a	3.55a
	Super Raya	2.24g	2.27g	2.22f	2.17ef	3.07c	3.10bc
	Rainbow	3.44b	3.55ab	3.14b	3.14a	3.62a	3.66a
CAT (U mg^−1^ protein)	Faisalabad Canola	0.32fg	0.30f	0.30f	0.35e	0.30cde	0.29cd
	Punjab Canola	0.28g	0.29f	0.29f	0.29ef	0.29de	0.29cd
	Dunkled	0.25g	0.28f	0.29f	0.24f	0.27e	0.27cd
	Oscar	0.41e	0.50bc	0.54bcde	0.61ab	0.40b	0.32c
	Legend	0.40ef	0.39e	0.51cde	0.55bcd	0.34bcde	0.26cd
	AARI Canola	0.42de	0.45cde	0.57abcd	0.61ab	0.36bc	0.29cd
	AC Exul	0.44de	0.48bcd	0.56abcd	0.58abc	0.36bcd	0.29cd
	CON-II	0.40ef	0.40e	0.49de	0.53cd	0.29de	0.23d
	CON-III	0.45cde	0.42de	0.48e	0.57abc	0.33bcde	0.25cd
	Cyclone	0.39ef	0.53ab	0.61ab	0.63a	0.36bcd	0.31c
	Super Canola	0.55ab	0.59a	0.57abc	0.57abc	0.59a	0.55b
	Sandal Canola	0.54ab	0.51abc	0.59ab	0.53cd	0.61a	0.57b
	Shiralaee	0.57a	0.58a	0.62a	0.63a	0.60a	0.66a
	Super Raya	0.52abc	0.53ab	0.55abcde	0.52cd	0.56a	0.56b
	Rainbow	0.50bcd	0.54ab	0.57abc	0.48d	0.54a	0.54b

Values are means of three replicates while different letters showed significant changes compared with Fisher LSD; p ≤ 0.05.

The results showed that the SOD activity varied significantly among the Canola varieties at different SW levels. At 20SW, the SOD activity of Faisalabad Canola decreased by 1.06%, while Punjab Canola showed a slight increase of 0.62%. Dunkled showed the maximum decrease of 8.33% in SOD activity compared to tap water. On the other hand, Oscar showed a substantial increase of 80.51%. Legend and AARI Canola showed a significant increase of 22.64% and 23.17%, respectively, compared to tap water at 20SW.

Similarly, AC Exul, Cyclone, and Sandal Canola also showed an increase in SOD activity at 20SW compared to tap water. At 40SW, the SOD activity of all Canola varieties decreased compared to tap water. The maximum decrease was observed in Oscar, with a decline of 45.89%, while Shiralaee showed the least decrease of 0.79%. At 60SW, the SOD activity of all Canola varieties decreased significantly compared to tap water, with Dunkled showing the maximum decrease of 10.07%. However, Oscar showed an increase of 80.51%, and Legend showed an increase of 26.42% compared to tap water at 60SW. At 80SW and 100SW, the SOD activity of all Canola varieties decreased substantially compared to tap water. The maximum decrease was observed in Dunkled at 80SW and 100SW, with a decline of 11.11% and 22.35%, respectively. Oscar decreased 64.75% and 73.77% at 80SW and 100SW, respectively, compared to tap water. Similarly, Super Canola and Sandal Canola also showed a considerable decrease in SOD activity at higher SW levels than tap water.

At 20SW, the MDA range from 1.09 to 3.46 nmol g^−1^ fresh weight, with Super Canola and Sandal Canola having the highest values. Compared to tap water, Super Canola and Sandal Canola exhibit an increase in MDA by 126% and 160%, respectively. Under 40SW, the MDA range from 1.05 to 3.51 nmol g^−1^ fresh weight, with Sandal Canola and Shiralaee having the highest values. Compared to tap water, Sandal Canola exhibits a slight increase in MDA by 0.6%, while Shiralaee exhibits an increase of 155%. For 60SW, the MDA range from 1.04 to 3.56 nmol g^−1^ fresh weight, with Shiralaee and Sandal Canola having the highest values. Compared to tap water, Shiralaee exhibits an increase in MDA by 155%, while Sandal Canola exhibits a decrease in MDA by 1.5%. In 80SW, the MDA range from 1.08 to 3.60 nmol g^−1^ fresh weight, with Shiralaee and Super Canola having the highest values. Compared to tap water, Shiralaee exhibits an increase in MDA by 154%, while Super Canola exhibits a decrease in MDA by 5.6%. At 100SW, the MDA range from 1.08 to 3.70 nmol g^−1^ fresh weight, with Shiralaee and Sandal Canola having the highest values. Compared to tap water, Shiralaee exhibits an increase in MDA of 148%, while Sandal Canola exhibits a decrease in MDA by 5.8% (Table 3).

**Table 3:** Effect of treatments on SOD and MDA of different canola cultivars.

Attribute	Varieties	Tap water	20SW	40SW	60SW	80SW	100SW
SOD (U mg^−1^ protein)	Faisalabad Canola	1.88h	1.86g	1.69f	1.75h	1.85fgh	1.79d
	Punjab Canola	1.61hi	1.62gh	1.48fg	1.52hi	1.57hi	1.53de
	Dunkled	1.44i	1.32h	1.29g	1.28i	1.31i	1.33e
	Oscar	2.44fg	3.39e	3.95cde	4.18f	1.76fgh	1.33e
	Legend	2.65f	3.26ef	4.20bc	4.81bcd	2.27e	1.41e
	AARI Canola	3.11e	3.82d	4.79a	5.20a	2.36de	1.62de
	AC Exul	3.45d	3.82d	4.30b	5.12ab	2.63d	1.54de
	CON-II	2.33g	2.97f	3.74e	4.51de	1.62ghi	1.48de
	CON-III	2.61fg	3.00f	3.82de	4.74cde	2.04ef	1.54de
	Cyclone	2.71f	3.40e	4.33b	5.00abc	1.92fg	1.50de
	Super Canola	4.33b	4.38c	4.37b	4.47ef	4.50b	4.55b
	Sandal Canola	4.60b	4.74b	4.80a	4.85bc	4.88a	4.98a
	Shiralaee	5.03a	5.07a	5.10a	5.17a	5.17a	5.23a
	Super Raya	3.99c	4.04d	4.09bcd	4.15fg	4.22bc	4.25bc
	Rainbow	3.75cd	3.81d	3.84de	3.85g	3.92c	3.95c
MDA (nmol g^−1^ fresh weight)	Faisalabad Canola	1.41k	1.36l	1.30m	1.29m	1.32l	1.29m
	Punjab Canola	1.25l	1.22m	1.18n	1.20n	1.19m	1.15n
	Dunkled	1.09m	1.05n	1.04o	1.08o	1.06n	1.08o
	Oscar	1.41k	1.50k	1.53l	1.51l	1.61k	1.62l
	Legend	1.65j	1.71j	1.74k	1.73k	1.77j	1.82k
	AARI Canola	1.86i	1.93i	1.97j	1.95j	2.04i	2.06j
	AC Exul	2.08h	2.05h	2.04i	2.11i	2.17h	2.15i
	CON-II	2.29g	2.28g	2.29h	2.28h	2.28g	2.27h
	CON-III	2.35g	2.39f	2.40g	2.50g	2.50f	2.51g
	Cyclone	2.48f	2.63e	2.60f	2.60f	2.69e	2.67f
	Super Canola	3.03c	3.09c	3.12c	3.14c	3.23b	3.24c
	Sandal Canola	3.28b	3.30b	3.35b	3.34b	3.30b	3.45b
	Shiralaee	3.46a	3.51a	3.56a	3.60a	3.56a	3.70a
	Super Raya	2.72e	2.70e	2.81e	2.79e	2.81d	2.80e
	Rainbow	2.91d	2.93d	2.91d	2.95d	3.08c	3.02d

Values are means of three replicates while different letters showed significant changes compared with Fisher LSD; p ≤ 0.05.

Regarding tap water, Faisalabad Canola had the highest chlorophyll a content at 1.10 mg g^−1^, while Dunkled had the lowest at 0.72 mg g^−1^. At 20SW, Faisalabad Canola had a slight increase in chlorophyll a content compared to tap water, with a value of 1.14 mg g^−1^. In contrast, Punjab Canola slightly decreased at 1.01 mg g^−1^. Dunkled also decreased at 0.74 mg g^−1^, the lowest value among the varieties. Oscar significantly decreased at 0.89 mg g^−1^, while Legend slightly increased at 0.72 mg g^−1^. AARI Canola and AC Exul had the highest chlorophyll a content at 1.08 mg g^−1^ and 1.25 mg g^−1^, respectively, indicating an increase in chlorophyll a content at 20SW. CON-II, CON-III, Cyclone, Super Canola, Sandal Canola, Shiralaee, and Super Raya all showed a decrease in chlorophyll a content at 20SW compared to tap water. Under 40SW, Faisalabad Canola continued to have the highest chlorophyll a content at 1.15 mg g^−1^, while Dunkled had the lowest at 0.67 mg g^−1^. Punjab Canola, Dunkled, and Super Canola all showed a decrease in chlorophyll a content compared to tap water. Oscar and Legend increased at 1.03 mg g^−1^ and 1.12 mg g^−1^, respectively. AARI Canola had a decrease in chlorophyll a content at 0.71 mg g^−1^. At the same time, AC Exul, CON-II, CON-III, Cyclone, Sandal Canola, Shiralaee, and Super Raya all showed an increase. For 60SW, Faisalabad Canola peaked in chlorophyll a content at 1.24 mg g^−1^, while Dunkled had the lowest at 0.62 mg g^−1^. Punjab Canola, Dunkled, and Super Canola all had a decrease in chlorophyll a content compared to tap water. Oscar, Legend, and AARI Canola all had an increase, while AC Exul, CON-II, CON-III, Cyclone, Sandal Canola, Shiralaee, and Super Raya all had a decrease in chlorophyll a content. In 80SW, Faisalabad Canola continued to have the highest chlorophyll a content at 1.25 mg g^−1^, while Dunkled had the lowest at 0.55 mg g^−1^. Punjab Canola, Dunkled, and Super Raya all showed a decrease in chlorophyll a content compared to tap water. Oscar and Legend had an increase, while AARI Canola, AC Exul, CON-II, CON-III, Cyclone, Sandal Canola, and Shiralaee all had a decrease in chlorophyll a content. For 100SW, Faisalabad Canola still had the highest chlorophyll a content at 1.35 mg g, while Super Raya had the lowest at 0.60 mg g^−1^. Punjab Canola, Dunkled, Super Canola, and Super Raya all had a decrease in chlorophyll a content compared to tap water. Similarly, for Dunkled variety, there was a slight increase in chlorophyll a content at 20SW and 40SW compared to tap water. However, at higher SW levels, there was a significant decrease. At 80SW and 100SW, the chlorophyll a content decreased by 14.3% and 23.6%, respectively. Oscar variety showed a mixed response to SW. At 20SW, the chlorophyll a content decreased by 20.2%, while at 40SW and 80SW, it increased significantly by 38.8% and 23.2%, respectively. However, at 60SW and 100SW, the chlorophyll a content decreased by 16.5% and 16.1%, respectively. In contrast, Legend variety showed a significant increase in chlorophyll a content with increasing SW levels. At 40SW, the chlorophyll a content increased by 30.2%, and at 80SW and 100SW, it increased by 17.4% and 16.3%, respectively. AARI Canola, AC Exul, and CON-III varieties also showed a similar pattern of increased chlorophyll a content at higher SW levels.

In the case of 20SW, it was observed that Faisalabad Canola, Dunkled, Oscar, and Sandal Canola had a significant increase in chlorophyll b content compared to tap water, with percentage increases of 60%, 47%, 38%, and 67%, respectively. On the other hand, Punjab Canola, Cyclone, AARI Canola, AC Exul, CON-III, and Rainbow showed a decrease in chlorophyll b content, with percentage decreases ranging from 11 to 36%. Legend, CON-II, Super Canola, Shiralaee, and Super Raya had no significant change in chlorophyll b content at this SW level. For 40SW, Faisalabad Canola, Cyclone, CON-III, and Super Raya exhibited a significant increase in chlorophyll b content, with percentage increases ranging from 31 to 81%. Punjab Canola, Dunkled, Oscar, Legend, AARI Canola, AC Exul, Super Canola, Sandal Canola, Shiralaee, and Rainbow had a decrease in chlorophyll b content, with percentage decreases ranging from 4 to 44%. Under 60SW, Faisalabad Canola, CON-III, Cyclone, and Super Raya significantly increased chlorophyll b content, with percentage increases ranging from 15 to 30%. Punjab Canola, Dunkled, Oscar, Legend, AARI Canola, AC Exul, Super Canola, Sandal Canola, Shiralaee, and Rainbow showed a decrease in chlorophyll b content, with percentage decreases ranging from 15 to 60%. At 80SW, Faisalabad Canola, CON-III, and Super Raya significantly increased chlorophyll b content, with percentage increases ranging from 13 to 19%. Punjab Canola, Dunkled, Oscar, Legend, AARI Canola, AC Exul, Super Canola, Sandal Canola, Cyclone, Shiralaee, and Rainbow showed a decrease in chlorophyll b content, with percentage decreases ranging from 20 to 48%. In 100SW, Faisalabad Canola, CON-III, and Super Raya significantly increased chlorophyll b content, with percentage increases ranging from 7 to 14%. Punjab Canola, Dunkled, Oscar, Legend, AARI Canola, AC Exul, Super Canola, Sandal Canola, Cyclone, Shiralaee, and Rainbow showed a decrease in chlorophyll b content, with percentage decreases ranging from 31 to 69%.

For 20SW, Faisalabad Canola, AARI Canola, and CON-III showed an increase in total chlorophyll content by 11.5%, 5.8%, and 1%, respectively, compared to tap water. Punjab Canola, Dunkled, Legend, Cyclone, Super Canola, Sandal Canola, Super Raya, and Rainbow, on the other hand, exhibited a decrease in total chlorophyll content by 3.3%, 34.1%, 3.8%, 12.4%, 13.4%, 12.7%, 6.6%, and 6%, respectively. At 40SW, Faisalabad Canola, Punjab Canola, and AC Exul increased total chlorophyll content by 11.6%, 5.6%, and 17.7%, respectively, compared to tap water. Dunkled, Oscar, Legend, AARI Canola, CON-II, CON-III, Cyclone, Super Canola, Sandal Canola, Shiralaee, Super Raya, and Rainbow exhibited a decrease in total chlorophyll content by 31.7%, 15.5%, 16.1%, 37.5%, 29.3%, 31.5%, 8.2%, 18.8%, 25.3%, 18.3%, 17.3%, and 24.8%, respectively. In the case of 60SW, only Faisalabad Canola exhibited an increase in total chlorophyll content by 7.3%, compared to tap water. All other cultivars exhibited a decrease in total chlorophyll content, with Punjab Canola, Dunkled, and Legend showing the most significant decrease of 43.3%, 57.2%, and 13.2%, respectively. Under 80SW, only Faisalabad Canola exhibited an increase in total chlorophyll content by 6.1%, compared to tap water. All other cultivars exhibited a decrease in total chlorophyll content, with Dunkled, Oscar, Legend, and AARI Canola showing the most significant decrease of 72.3%, 64.3%, 35.8%, and 42.4%, respectively. For 100SW, only Faisalabad Canola exhibited an increase in total chlorophyll content by 4.8%, compared to tap water. All other cultivars exhibited a decrease in total chlorophyll content, with Dunkled, Oscar, Legend, AARI Canola, CON-II, Cyclone, Super Canola, Sandal Canola, Shiralaee, Super Raya, and Rainbow showing the most significant decrease of 91.3%, 87.3%, 54.9%, 71.2%, 81.2%, 53.3%, 43.7%, 57.2%, 59.3%, 81.8%, 52.3%, and 68.3%, respectively (Table 4).

**Table 4:** Effect of treatments on chlorophyll a, chlorophyll b and total chlorophyll of different canola cultivars.

Attribute	Varieties	Tap water	20SW	40SW	60SW	80SW	100SW
Chlorophyll a (mg g^−1^)	Faisalabad Canola	1.10a	1.14a–c	1.15a	1.24a	1.25a	1.35a
	Punjab Canola	1.07ab	1.01a–e	0.88a–e	0.85c–g	0.82d–f	0.78d–f
	Dunkled	0.72c	0.74ef	0.67e	0.62g	0.62f	0.55f
	Oscar	1.12a	0.89b–f	1.03a–d	0.93a–g	1.11a–d	0.94b–d
	Legend	0.86a–c	0.72ef	1.12ab	1.08a–d	1.01a–e	1.00b–d
	AARI Canola	0.94a–c	1.08a–d	0.71de	1.11a–c	0.92b–f	0.91b–e
	AC Exul	0.89a–c	1.25a	0.96a–e	1.12a–c	1.00a–e	1.24ab
	CON-II	0.89a–c	0.85c–f	0.96a–e	1.04a–e	1.09a–d	0.78d–f
	CON-III	0.95a–c	0.99a–f	0.92a–e	1.20ab	1.17a–c	1.12a–c
	Cyclone	0.82a–c	1.19ab	0.72de	0.88b–g	1.23ab	1.17a–c
	Super Canola	0.97a–c	0.79d–f	0.78c–e	0.77d–g	1.09a–d	0.96b–d
	Sandal Canola	0.90a–c	0.74ef	1.07a–c	0.69fg	1.01a–e	0.86c–f
	Shiralaee	0.74Bc	0.78d–f	0.81b–e	0.83c–g	0.93a–f	0.99b–d
	Super Raya	0.91a–c	0.87b–f	0.92a–e	0.71e–g	0.86c–f	0.60ef
	Rainbow	0.92a–c	0.66f	0.97a–e	0.98a–f	0.69ef	0.86c–f
Chlorophyll b (mg g^−1^)	Faisalabad Canola	0.56ab	0.34cd	0.68a	0.56a–d	0.43b	0.61ab
	Punjab Canola	0.36b	0.32d	0.64ab	0.57a–d	0.45b	0.47a–d
	Dunkled	0.51ab	0.34cd	0.64ab	0.66a	0.48b	0.39cd
	Oscar	0.53ab	0.51a–d	0.45b–d	0.60a–c	0.44b	0.59a–c
	Legend	0.47ab	0.48a–d	0.44b–d	0.55a–d	0.63ab	0.37d
	AARI Canola	0.46ab	0.39b–d	0.39cd	0.58a–d	0.45b	0.46a–d
	AC Exul	0.52ab	0.40b–d	0.52a–d	0.42b–d	0.42b	0.60a–c
	CON-II	0.61a	0.58ab	0.53a–d	0.62ab	0.53ab	0.62ab
	CON-III	0.51ab	0.48a–d	0.63ab	0.55a–d	0.61ab	0.56a–d
	Cyclone	0.51ab	0.39b–d	0.63ab	0.53a–d	0.53ab	0.43b–d
	Super Canola	0.52ab	0.66a	0.36d	0.44b–d	0.43b	0.53a–d
	Sandal Canola	0.60a	0.57ab	0.40cd	0.40cd	0.52ab	0.57a–d
	Shiralaee	0.46ab	0.63a	0.64ab	0.45a–d	0.45b	0.55a–d
	Super Raya	0.45ab	0.55ab	0.51a–d	0.53a–d	0.70a	0.66a
	Rainbow	0.37b	0.54a–c	0.58a–c	0.37d	0.46b	0.50a–d
Total chlorophyll (mg g^−1^)	Faisalabad Canola	1.66a	1.47ab	1.83a	1.80a	1.68ab	1.97a
	Punjab Canola	1.42ab	1.34a–c	1.52a–c	1.42a–f	1.27c–e	1.25de
	Dunkled	1.23b	1.08c	1.31b–d	1.29c–f	1.10e	0.94e
	Oscar	1.65a	1.39a–c	1.49a–d	1.53a–e	1.54a–d	1.53b–d
	Legend	1.33ab	1.20bc	1.55ab	1.63a–d	1.65a–c	1.36cd
	AARI Canola	1.39ab	1.46a–c	1.11d	1.70ab	1.36b–e	1.37cd
	AC Exul	1.41ab	1.66a	1.48a–d	1.55a–e	1.43a–e	1.83ab
	CON-II	1.50ab	1.43a–c	1.49a–d	1.67a–c	1.62a–c	1.39cd
	CON-III	1.45ab	1.47a–c	1.55ab	1.76a	1.79a	1.68a–c
	Cyclone	1.33ab	1.60a	1.36b–d	1.42a–f	1.77a	1.62a–d
	Super Canola	1.49ab	1.45a–c	1.15cd	1.20ef	1.53a–d	1.49b–d
	Sandal Canola	1.50ab	1.31a–c	1.46a–d	1.09f	1.55a–c	1.43cd
	Shiralaee	1.20b	1.42a–c	1.45a–d	1.27d–f	1.37b–e	1.54b–d
	Super Raya	1.37ab	1.41a–c	1.43b–d	1.24ef	1.55a–c	1.26de
	Rainbow	1.29ab	1.20bc	1.55ab	1.36b–f	1.16de	1.36cd

Values are means of three replicates while different letters showed significant changes compared with Fisher LSD; p ≤ 0.05.

## Discussion

In current study, results showed that 60% SW caused significant enhancement in canola growth attributes especially in Dunkled and Punjab Canola. The improvement in growth attributes might be due to enrichment of N, P and K in the SW. Secondly, dilution of SW also minimizes the toxic effects of heavy metals that eventually play an important role in reducing growth attributes and minimizing antioxidants in the canola. The presence of elevated levels of potassium and nitrogen in municipal wastewater has been observed to positively affect the growth of canola in hydroponic experiments^29,30^.

The high concentration of nitrogen in sugarcane industry effluent can promote the amino acids synthesis in plant, which are the building blocks of proteins and additionally can support chlorophyll formation, which is necessary for photosynthesis. Additionally, high nitrogen uptake by plants can increase nucleic acid synthesis, which are critical for DNA replication and cell division. The presence of high phosphorus in sugarcane industrial effluent** (**SW) can promote high uptake of phosphorus in plant can promote plant growth by enhancing root development, increasing photosynthesis, and stimulating the production of essential proteins and enzymes.

High productivity of crop in current study might by due to high potassium uptake due to potassium enriched industry effluent that helped plant to regulate various plant functions such as water balance, stomatal opening and closing, and photosynthesis^31^. The activation of approximately 60 enzymes in the plant system depends on potassium, which plays a crucial role as a regulator^31^. The enrichment of nitrogen, potassium, and phosphorus in municipal wastewater can be seen as a positive influence on crop growth in current study^32,33^.

The presence of magnesium and calcium in sugarcane effluent also positively impacted the growth of canola seedlings. The high uptake of magnesium, can promote chlorophyll formation which could increase rate of photosynthesis, and high magnesium might be increased activation of various enzymes and the regulation of plant metabolism^34^. High calcium uptake in plants can serve as a signaling molecule, regulating cell division and elongation, root and shoot development, and cell wall formation. Therefore, high uptake of calcium and magnesium due to enriched sugarcane effluent can strengthening the plant structure, calcium helps to enhance the seedlings' ability to resist environmental stressors, both biotic and abiotic^34^.

Low antioxidant production in resistant canola cultivars under municipal waste water treatment can indicate stress tolerance in these varieties^35^. In these cultivars, the plant may have developed alternative mechanisms to deal with the oxidative stress caused by the presence of heavy metals and other pollutants in the municipal waste water^35^. This, in turn, leads to lower antioxidant production, a common response to reduced levels of oxidative stress^35^.

The high uptake of nitrogen, phosphorus, and potassium might be promoted better growth and higher biomass production in plants^36^. However, higher levels of these nutrients can also suppress the production of antioxidants, as plants allocate more resources towards growth^34^. Therefore, by applying the nutrient enriched industrial effluent and high plant uptake, plants can increase the synthesis of various metabolic compounds, leading to increased shoot and root growth and fresh and dry weight^34^.

The current study primarily focuses on the positive effects of SW enriched with nutrients on canola growth attributes, specifically Dunkled and Punjab Canola. While the study highlights the benefits of nutrient enrichment and dilution of SW in enhancing growth, it does not extensively explore the potential negative consequences or trade-offs associated with these practices. Specifically, the study mentions suppressing antioxidant production in response to high nutrient levels, which could indicate stress tolerance in canola cultivars. However, it does not delve into the implications of reduced antioxidant production, such as potential impacts on plant health, resilience to pests and diseases, or overall crop quality. Moreover, the study does not investigate the long-term effects of nutrient-rich SW on soil quality, water resources, or the environment, which is crucial for assessing the sustainability of such irrigation practices.

Future research in this domain should prioritize perfecting nutrient management strategies, aiming to maximize the advantages of utilizing nutrient-rich wastewater for irrigation while also addressing potential drawbacks, such as the possible reduction in antioxidant production. Achieving this goal involves adjusting nutrient concentrations in irrigation water tailored to specific crop varieties, investigating inventive wastewater treatment methods to boost nutrient levels and diminish heavy metal contaminants, and undertaking comparative investigations to understand how different crops respond to this irrigation approach. This research holds significant importance in advancing sustainable agricultural practices that optimize the utilization of available resources, all while safeguarding crop well-being and yield in the context of mounting challenges related to water scarcity and environmental concerns.

## Conclusion

In conclusion, the current study highlights the substantial benefits of using nutrient-enriched municipal wastewater for canola irrigation, particularly in the case of Dunkled and Punjab Canola. Nutrient enrichment, including nitrogen, phosphorus, potassium, magnesium, and calcium, significantly improved canola growth attributes, enhancing root development, photosynthesis, and overall biomass production. However, it's crucial to recognize that the study primarily emphasizes the positive outcomes and does not delve deeply into potential trade-offs, such as reduced antioxidant production and long-term environmental impacts. Nevertheless, further investigation is needed to fully understand the broader implications and fine-tune the application of this practice for maximizing agricultural benefits while minimizing potential negative consequences.

## Data Availability

All data generated or analysed during this study are included in this published article.
